# Systematic review of initiatives promoting health career paths for secondary-level students

**DOI:** 10.3389/phrs.2026.1609646

**Published:** 2026-07-02

**Authors:** Julia Stiz, Anne-Linda Camerini

**Affiliations:** 1 Department of Paediatrics, Cumming School of Medicine, University of Calgary, Calgary, AB, Canada; 2 Faculty of Biomedical Sciences, Institute of Public Health, Universita della Svizzera Italiana, Lugano, Switzerland

**Keywords:** careers, healthcare, interventions, secondary, students, systematic review

## Abstract

**Objectives:**

This systematic review examined healthcare career promotional initiatives targeting secondary-level students to characterize initiative types, evaluate their effectiveness at influencing students’ knowledge, interest, attitudes, and healthcare career pathways, and identify factors influencing program success.

**Methods:**

A pre-registered systematic review was conducted following PRISMA guidelines. Seven databases were searched. Analysis and interpretation were guided by a modified Motivation–Opportunity–Ability (MOA) model. Study quality was assessed with adapted checklists.

**Results:**

Fifty studies with a total of 5,315 participants were included. Forty-four initiatives were North American, targeting grades eleven and twelve through camps, courses, internships, mentorships, and continuum programs. Studies observed short-term improvements in interest, attitudes, knowledge, and career pathways. The success of initiatives was associated with flexibility, accessibility, funding, cultural relevance, and community involvement. Risk of bias assessment was heterogeneous, ranging from moderate to high.

**Conclusion:**

Current healthcare career promotional initiatives are promising but constrained by moderate quality, geographical limitation, and heterogeneity. *The Integrated MOA-Informed Model* offers a valuable framework for the design and evaluation of future initiatives, highlighting the need for more rigorous, diverse, and longitudinal evaluations.

**Systematic Review Registration:**

https://www.crd.york.ac.uk/PROSPERO/view/CRD42024515728, identifier CRD42024515728.

## Introduction

Over time, the healthcare workforce has undergone significant changes due to demographic shifts, technological advancements expanding the roles of traditional healthcare, and disease burdens [1]. These changes have resulted in the healthcare workforce facing persistent shortages, with the World Health Organization (WHO) warning of a shortage of 10 million healthcare workers by 2030 [2]. Aging healthcare workers, high turnover rates, and chronic underinvestment in education further exacerbate workforce challenges [2–4]. These shortages have serious repercussions, impacting health outcomes, increasing costs, and exacerbating disparities, especially in rural and underrepresented populations of increasingly diversifying nations as well as low-middle income countries as high-income countries look to these nations to recruit healthcare workers [5–7].

This overview of the current situation of healthcare workforce highlights the need for broad strategies to address the healthcare workforce shortage. Initiatives promoting early recruitment and engagement are considered medium to long term strategies. However, they have been overlooked in recent years as there has been a shift in policy focus to healthcare worker retention due to its more immediate effects [8]. Evidence has shown that domestic recruitment efforts do work but require investment and time [3]. Sustaining the number of students admitted into healthcare profession schools is key to avoiding future shortages [8]. Policy decisions need to align with a robust approach to healthcare workforce development, not solely focusing on retention. To achieve this, healthcare careers—particularly those in direct patient care roles—need to be promoted at an early age. Direct patient care roles include various clinical care professions involved in diagnosis, treatment, and management of medical conditions. They also include various allied health professions that support and complement the work of clinical professionals, often through specialized technical or therapeutic services that contribute to patient care. For example, medicine, nursing, allied health professions such as physical or occupation therapists, pharmacist, radiographers, as well as community health workers such as emergency medical technicians and certified nursing assistants or healthcare aides all are considered direct patient care careers.

Promotion of direct patient care healthcare careers to secondary-level students is a promising strategy to increase the future healthcare workforce. Understanding youth career development in this context requires a multifaceted approach that considers both intrinsic factors, such as personal interest, and extrinsic factors, including job security, remuneration, and family influence [9]. Negative perceptions, limited awareness, and insufficient exposure to direct patient care roles, particularly in rural and underrepresented communities, can hinder career interest [10, 11].

Given the complexity of youth career development, established theoretical frameworks provide a robust foundation for understanding how initiatives can shape knowledge, interest, and attitudes. Ginzberg’s General Theory of Occupational Choice [12] highlights developmental stages in career decision-making, while Super’s Developmental Self-Concept Theory [13, 14] emphasizes evolving self-concepts, or how individuals perceive their abilities, interests, and identity in relation to careers, across the lifespan. Bronfenbrenner’s Socio-Ecological Model (SEM) [15] situates career development within multiple interacting environmental systems, such as schools, families, and broader social contexts. Adaptations of models like the Motivation–Opportunity–Ability (MOA) Model [16] to career promotion contexts further demonstrate the value of theory-informed approaches, offering a clear structure for interpreting how opportunities influence youth motivation and ability, which in turn influence career behaviour. The MOA model is a behavioral model originally conceptualized for marketing and understanding consumer behavior but is adaptable and applicable to various contexts. The model posits that an individual’s behavior is primarily influenced by personal attributes, such as motivation and ability, as well as external factors like opportunities available in their environment [17]. It has been applied to medical students and the development of their occupational identity, in which it was found that medical students’ occupational identity is shaped by motivation, opportunity and ability factors [18].

Although there is substantial literature on the impact of broad career education on career choice [19–22] relatively little attention has been given specifically to careers in healthcare. Reviews in the realm of youth healthcare career promotion have primarily focused on single professional domains (e.g., nursing) [23], are non-specific to direct patient care roles [24] or have been limited to specific populations (e.g., Indigenous populations) [24, 25]. None have comprehensively explored promotional activities targeting all secondary-level students or have systematically assessed the effectiveness of initiatives at enhancing knowledge, interest, and attitudes toward a wide range of direct patient care roles or the career pathways pursued. Career pathways refer to the aspirations and trajectory participants follow in their career choices and educational pursuit, which includes the career path being considered, chosen career path, educational pathways, and the factors influencing career decisions and pathways.

This systematic review addresses these gaps by examining a diverse array of healthcare career promotion initiatives targeting secondary-level students, regardless of demographic background. The overarching aim is to identify best practices and contextual factors that contribute to initiatives’ success in fostering engagement with direct patient care careers among secondary-level students. To achieve this, the review is guided by the following research questions:

RQ1: Which initiatives have been implemented to promote direct patient care healthcare career paths among secondary-level students, and how do they differ in terms of target groups, goals, formats, features, and content?
RQ2: What is the effectiveness of these initiatives in terms of increasing knowledge, fostering interest, changing attitudes, and influencing career pathways?
RQ3: What are the contextual factors that influence the success of these initiatives?

By systematically addressing these questions, this review aims to provide a comprehensive understanding of effective initiatives in promoting direct patient care healthcare careers among youth. It will highlight the strengths and gaps in existing initiatives, offering insights into how future initiatives can be designed to maximize their impact.

**FIGURE 1:**
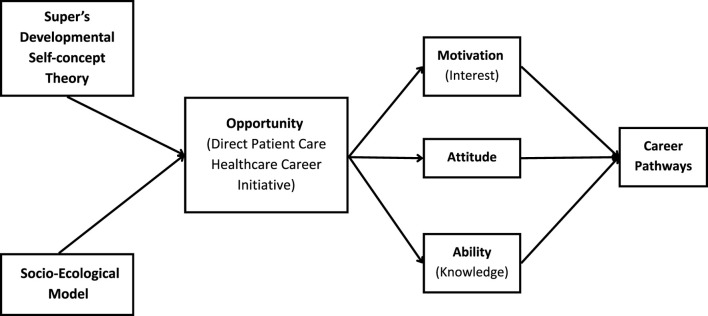
Adapted MOA Model (Lugano, Switzerland, 2024). Presents the Adapted Motivation–Opportunity–Ability model used to guide the analysis.

To guide the analysis of the review findings, an *Adapted MOA Model* (Figure 1) was developed. This model helps to illustrate how opportunity (direct patient care healthcare career initiatives) influences youth motivation (interest), attitudes, and ability (knowledge), which collectively influence healthcare career pathways, offering a clear framework for interpreting the data and presenting the studies’ outcomes.

## Methods

### Search strategy

The systematic review followed PRISMA guidelines [26] and was preregistered in PROSPERO (BLINDED FOR REVIEW) on 03.05.2024. Searches were conducted on 22.01.2024 across seven databases. The initial search included title and abstract searches, however, due to the overwhelming volume of studies returned by database queries, searches in EBSCOhost, ProQuest, and Web of Science were limited to titles, while PubMed included both title and abstract searches. This method aimed to obtain balance between comprehensiveness and feasibility, ensuring the search process remained manageable while still capturing pertinent studies relevant to the review’s objectives. According to the Population, Intervention, Comparison, Outcome (PICO) framework [27] keywords covered the *population* (e.g., secondary school, high school), *intervention* (e.g., education, promotion) in regard to the context (e.g., health, medical). *Comparison* and *outcome* were not defined to include interventions of different designs beyond controlled trials and addressing different outcomes to be grouped qualitatively in the qualitative synthesis. A manual citation search of relevant reviews was also performed to identify additional sources. Supplementary Material A includes the search string for each database.

### Eligibility criteria

Studies were included if they: (1) reported on planned healthcare career promotional activities for secondary school students, (2) focused on participants aged 12–18, (3) were published between 2013–2024, (4) peer-reviewed journal publications, and (5) examined knowledge, interest, attitudes, career pathways, or related concepts. Exclusion criteria included: (6) lack of explicit reporting on relevant outcomes, (7) non-career interventions, (8) focus on non-secondary school populations, (9) mixed-age participant groups without separation, (10) primary focus on academic preparation, (11) non-direct patient care healthcare career focus, (12) non-English full text, and (13) multimedia publications, theses, protocols, conference proceedings, book chapters, and reviews. Only promotional initiatives for direct patient care careers were considered, excluding healthcare research careers. For example, initiatives promoting careers in cancer research would be excluded whereas initiatives promoting a career as an oncologist would be included. Science, technology, engineering and math (STEM) initiatives were excluded unless explicitly referencing direct patient care careers. Academic preparatory programs were also excluded unless they served as a supplementary component rather than the primary focus.

### Study selection

Results from databases and citation searches were screened with a two-phase process. The initial phase consisted of title and abstract screening. Papers retained in this phase were then assessed for full-text screening. Among the full-text articles, only those meeting the eligibility criteria were included. The entire selection process was completed by the first author. In select cases, where the inclusion status of a study was not clear, consensus was achieved via consultation with the second author. The selection process was performed in the time frame from 02.02.2024 to 01.04.2024.

### Data collection

For each of the studies eligible for the systematic review, the following information were collected and reported into an Excel file: author’s name, year of publication, initiative name, goal/objective of the initiative, study design, sample, target population, theory, initiative provider, initiative description (format, special design features, setting, duration, activities, planning and preparation, funding) and evaluation (outcome of interest, methods and results). Results were coded whether the estimated overall effect increased (+), had no effect (N.S), or decreased (−).

Once collected, all data were reviewed and categorized. Outcomes were categorized into one or more of the predefined categories in line with the *Adapted MOA model*, including knowledge, interest, attitudes, and career pathways. Initiative formats were categorized into eight broad types guided by the following definitions: (1) afterschool program (i.e., a series of structured sessions specifically designed to support students’ exploration of healthcare careers outside regular school curriculum and regular school hours), (2) course (i.e., formal academic course with defined learning objectives and assessment), (3) enrichment camp (i.e., a short-term, immersive experience designed to introduce student participants to various aspects of healthcare professions), (4) exposure event (i.e., a structured event exposing students to different healthcare careers, settings, roles and activities), (5) exposure program (i.e., a series of intermittent events exposing students to different healthcare careers, settings, roles and activities), (6) internship (i.e., hands-on experience in a professional healthcare setting), (7) mentorship (i.e., pairing students with healthcare representatives to provide guidance, support and insights), (8) continuum (i.e., a multi-session, continuous support program).

### Risk of bias assessment

To assess the risk of bias, modified versions of established checklists were used, including adaptations of Consolidated Criteria for Reporting Qualitative Research (COREQ) [28], Case Report (CARE) guidelines [29], and the Strengthening the Reporting of Observational Studies in Epidemiology (STROBE) checklist [30]. Adjustments were made to fit the context of healthcare career promotion initiatives for secondary students. Each study was graded based on relevant checklist criteria, with scores converted to a 0–1 scale for cross-methodological comparison. Studies were categorized as high (0.75–1.0), moderate (0.50–0.74), or poor quality (0–0.49) based on methodological rigor and risk of bias, following Cochrane Handbook guidelines [31]. The adapted checklist is in Supplementary Material B.

## Results

**FIGURE 2:**
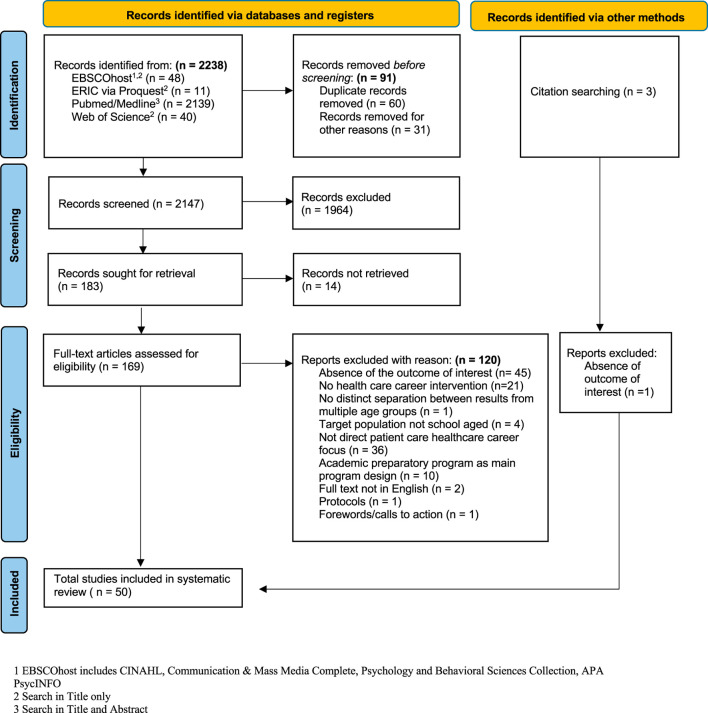
Preferred Reporting Items for Systematic Reviews and Meta-Analyses *flow chart (Lugano, Switzerland, 2024)*. Presents the Preferred Reporting Items for Systematic Reviews and Meta-Analyses flow diagram outlining the study identification, screening, eligibility assessment, and inclusion process for the systematic review.

The search strategy identified 2,238 records. After removing 60 duplicates and 31 ineligible publication formats (i.e., not peer-reviewed), 2,147 abstracts were assessed, with 1964 excluded during title and abstract screening. Of the remaining 183 full-text articles, 14 were unavailable, and 120 were excluded. Three additional articles were identified through reference screening, with one later excluded, leaving 50 articles reporting on 50 studies for inclusion (see Figure 2 for the complete Preferred Reporting Items for Systematic Reviews and Meta-Analyses (PRISMA) flowchart).

**FIGURE 3:**
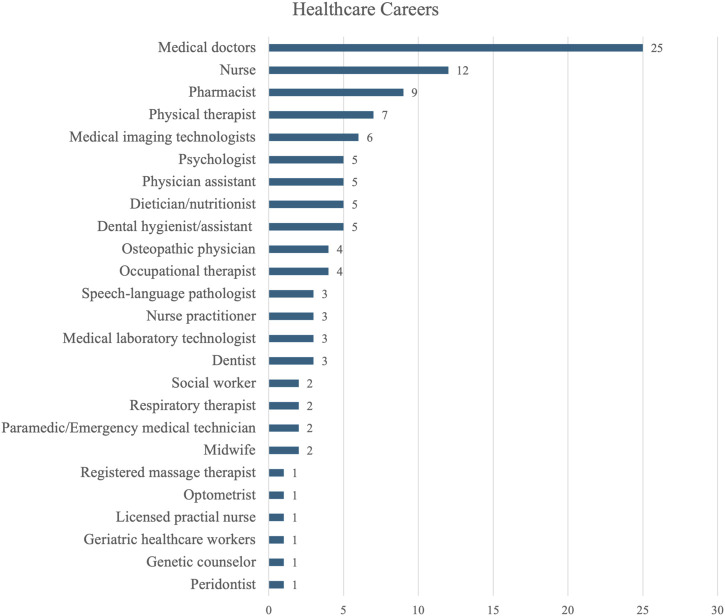
Frequency of healthcare careers referenced in included publications (Lugano, Switzerland, 2024). Presents the healthcare careers promoted across the included publications and the frequency with which each career was represented.

Table 1 provides a summary of the included studies. Most evaluation studies were quantitative (33, 66%), with fewer qualitative (3, 6%) and mixed-methods (14, 28%) approaches. Nearly half (23, 46%) were descriptive, while 27 (54%) included inferential analysis. Most initiatives were carried out in North America (44, 88%), primarily addressing healthcare workforce diversity (27, 54%) or rural healthcare workforce development (12, 24%). United States (U.S.) school grading was used [7th (12–13 years), 8th (13–14 years), 9th/Freshman (14–15 years), 10th/Sophomore (15–16 years), 11th/Junior (16–17 years), and 12th/Senior (17–18 years)], with 11th/12th grades most targeted. Common formats included exposure events (15, 30%), enrichment camps (13, 26%), and afterschool programs (9, 18%). The most cited healthcare careers were medical doctors (25, 50%), nurses (12, 24%), pharmacists (9, 18%) and dentists (9, 18%). The different careers and the frequency at which they were mentioned can be found in Figure 3. Detailed summaries of initiative characteristics, including program structure, delivery format, setting, and coordinating organizations, as well as activities included in each initiative can be found in Supplementary Materials C,D.

**TABLE 1:** Summary of included publications (Lugano, Switzerland, 2024).

Author, year	Program name	Country	Target POP’L	Education level	Program format	Sample (N)	Study design	Outcomes	Results				Quality check score
									I	A	K	CP	
Atance [32]	Summer enrichment experience (SEE)	US	Rural	10–12	ENR CAMP	45	Quan	I, A, K	+/n	+/n	+	​	0.75
Banuelos [33]	Summer premed program	US	URiM	10–12	ENR CAMP	371	Mixed methods	I	+	​	​	​	0.69
Berk [34]	MEDscience	US	•	HS	Course	30	Quan	I, A, CP	+	+	​	+	0.92
Briskey [35]	DUCOM mini-medical school	US	•	12	ENR CAMP	30	Quan	I, K	ns	​	+	​	0.89
Burns [36]	Saturday academy	US	URiM	HS	ASP	55	Quan	I, CP	+	​	​	+	0.78
Burns [37]	Saturday academy	US	URiM	HS	ASP	60	Quan	I	+	​	​	​	0.89
Butler [38]	Healthcare diversity summer camp	US	URiM	10–12	ENR CAMP	107	Quan	I, K, CP	+	​	+	+	0.86
Coffin [39]	Perry outreach program	US	URiM	HS	EXP EVENT	18	Quan	I, A	+	+	​	​	0.78
Crawford [40]	Programme incubator	NZ	URiM	10–12	EXP PROG	92	Quan	I	+	​	​	​	0.86
Crump [41]	High school rural scholar (HSRS)	US	Rural	12	ENR CAMP	151	Quan	I, A, K, CP	ns	+	+	+	0.61
D'anna [42]	Health sciences careers dual credit program	US	•	HS	Course	5,315	Quan	CP	​	​	​	+	0.82
Das [43]	Doctors day	US	URiM, rural	HS	EXP EVENT	91	Quan	I, K	+	​	+	​	0.77
Dicosmo [44]	Inspiring Women in orthopedics And engineering (IWOAE)	US	URiM	10–12	EXP EVENT	475	Quan	I, K	+	​	+	​	0.77
Fernandez-Repolle [45]	Summer health science career internship	Puerto Rico	URiM	MS, HS	ENR CAMP	49	Quan	I, K	+	​	+	​	0.71
Freischlag [46]	Health career academy (HCA)	US	URiM	10–11	ASP	36	Quan	I, K	+	​	+	​	0.64
Frey [47]	Brain bee	US	•	HS	EXP EVENT	34	Mixed methods	I	+	​	​	​	0.78
Gefter [48]	Health career academy (HCA)	US	URiM	10–12	CTM	292	Mixed methods	I, K	+	​	+	​	0.83
Ghazali [49]	Pharmacy Awareness campaign	Nigeria	•	10–12	EXP EVENT	127	Quantitative	I, K	+	​	+	​	0.73
Goldsmith [50]	•	US	•	7th	EXP EVENT	53	Quan	I, K	+	​	+	​	0.77
Gómez [51]	Student career opportunity outreach program (SCOOP)	US	•	HS	INTERN	84	Quan	I, CP	+	​	​	+	0.68
Hamrick [52]	Just say know to drugs!	US	Rural	9–12	ENR CAMP	37	Quan	I, K	+	​	+	​	0.71
Henderson [53]	CSM mini-med school (MMS)	Canada	URiM	7–12	EXP PROG	49	Quan	I	+	​	​	​	0.57
Herek [54]	Yes: Cancer biology And you day	US	URiM	HS	EXP EVENT	81	Mixed methods	I, K	+	​	+	​	0.76
Holden [55]	Mentoring in medicine After school program (MIM-ASP)	US	URiM	9–12	ASP	84	Mixed methods	I, A, K, CP	+	+	+	+	0.74
Holden [56]	Mentoring in medicine virtual science camp (MIM-VSC)	US	URiM	HS, college	ENR CAMP	55	Quan	I, K	+	​	+	​	0.63
Inglehart [57]	Ypsilanti high school recruitment through engagement program	US	URiM	9–12	ASP	50	Quan	I, K	+	​	+	​	0.77
Kadavakollu [58]	ACT And biomedical sciences enrichment program	US	Rural	9–12	ENR CAMP	82	Quan	I, K, CP	+	​	+	+	0.93
Karpa [59]	PULSE	US	•	HS	ASP	45	Quan	CP	​	​	​	+	0.73
Kaye [60]	High school mini-medical school program	US	Rural	10–12	EXP EVENT	69	Quan	I, K	+	​	+	​	0.70
Kendrick [61]	Medical education resources initiative for teens (MERIT)	US	URiM	11th	INTERN	65	Quan	I, A	ns	+	​	​	0.70
Keselman [62]	Teen health leadership program (THLP)	US	URiM	11–12	ASP	11	Qual	K, CP	​	​	+	+	0.60
Keselman [63]	Project SHARE: Student health advocates redefining empowerment	US	URiM	•	ASP	81	Mixed methods	I, K	+/n	​	ns	​	0.61
Kohut [64]	Lang youth medical program (LYMP)	US	URiM	7th	CTM	27	Qual	A, K, CP	​	+	+	+	0.88
Kumar [65]	The broken hill regional health career academy program (BHRHCAP)	Australia	Rural	7–12	EXP EVENT	33	Qual	I, CP	+	​	​	+	0.74
Labadie [66]	Summer surgery program (SSP)	US	•	HS	ENR CAMP	77	Quan	I, K, CP	+	​	+	+	0.70
Macaskill [67]	Aspire2Health	Australia	Rural	9–10	EXP EVENT	373	Mixed methods	I, K	+	​	+	​	0.85
Maurice [68]	Healthcare travelling roadshow	Canada	Rural	10th	EXP EVENT	22	Mixed methods	I	+	​	​	​	0.83
Mayberry [69]	Urban impressions (UI) and dental imprint (DI)	US	URiM	7–8	EXP PROG	UI: 86 DI: 69	Quan	I, K	+	​	+	​	0.66
Oshiro [70]	Rural eMentoring BC	Canada	Rural	7–12	MENTOR	209	Mixed methods	I, A	+	ns	​	​	0.85
Patel [71]	Dixie AHEC scholars program (DASP)	US	URiM, rural	9–12	EXP PROG	141	Quan	K	​	​	ns	​	0.54
Patel [72]	Medical student mentorship program	US	URiM	11–12	MENTOR	15	Quan	I, K, CP	+	​	+	+	0.73
Pezzullo [73]	Saturday academy	US	URiM	11–12	ASP	172	Quan	I, CP	+	​	​	+	0.75
Pruszynski [74]	Mayo clinic career Advancement, research, And education summer (CARES)	US	URiM	9–11	ENR CAMP	38	Mixed methods	K	​	​	+	​	0.47
Robinson [75]	Southwestern Ontario medical mentorship program (SWOMMP)	Canada	Rural	10–12	EXP EVENT	45	Mixed methods	I, A	+	+	​	​	0.62
Rocha [76]	Health science teaching excellence program (H-STEP)	US	URiM	9th	ENR CAMP	46	Mixed methods	I, CP	+	​	​	+	0.60
Rodriguez [77]	Mobilizing physician Assistants: Educational And professional outreach to underserved urban communities (MPA) program	US	URiM	10–12	EXP PROG	24	Mixed methods	I, K	-	​	+	​	0.88
Rogers [78]	•	US	•	HS	EXP EVENT	31	Quan	I, K	ns	​	+	​	0.79
Shaikh [79]	Mini-medical school (MMS)	Ireland	Rural	10th	ENR CAMP	90	Quan	I, K	+	​	+	​	0.61
Tawash [80]	NURS‐P.R.A.M Nursing - positive recruitment Arabic model	Bahrain	•	12th	EXP EVENT	72	Mixed methods	I, K	+	​	+	​	0.65
Zhang [81]	Anatomy And pathology Workshop	US	•	12th	EXP EVENT	128	Quan	I, K	+	​	+	​	0.70

*ENR, Camp = enrichment camp, EXP, event = exposure event, EXP PROG, exposure program; ASP, afterschool program; MENTOR, mentorship; CTM, continuum; INTERN, internship.

*URiM = under represented in medicine.

*Quan = quantitative, Qual = qualitative.

*I = interest, A = attitude, K = knowledge, CP = career pathways.

*ns = non-significant, +/n = significant and non-significant.

*HS, highschool; MS, middle school.

*Sections with “•” indicate no information was provided.

Table 1 presents a summary of the characteristics of the included studies, including program and participant characteristics, study design, outcome domains assessed (knowledge, attitudes, interest, and career pathways), and quality appraisal scores.

Interest was the most frequently examined outcome (44, 88%), followed by knowledge (32, 64%), career pathways (32, 64%), and attitude (9, 18%). Eighty percent of studies assessed multiple outcomes, with interest and knowledge often studied together, while only 20% focused on one outcome. Overall, initiatives improved interest in thirty-nine out of forty-four studies (89%), attitudes in eight out of nine studies (89%), knowledge in thirty out of thirty-two studies (94%), and the choice of a career pathway after secondary school in sixteen out of sixteen studies (100%).

The impact of initiatives did not vary considerably depending on the objective or format. Continuum programs and structured courses consistently demonstrated positive effects across all outcomes. In contrast, enrichment camps reported the highest proportion of non-significant findings, particularly for interest, with three of 13 studies (23%) showing no significant post-program changes. The characteristics of initiatives also influenced outcomes differently. Initiatives with a singular healthcare career focus were more likely to report non-significant gains in interest, whereas those designed around disease-specific content, such as cancer, consistently yielded positive effects across all outcomes. Similarly, body system-based programs showed positive results across most outcomes, except for interest in two studies. Body-system approaches refer to those focusing on one or multiple body systems and discuss careers associated with each system (e.g., cardiovascular system would teach about cardiac technicians, echocardiographers, cardiologists etc.), whereas those focusing on a single healthcare career would discuss only a career in e.g., nursing.

Study quality ranged from 0.47 to 0.93, with 48% [24] rated high, 50% [25] moderate, and one study poor. Most publications were thorough in abstracts, introductions, program descriptions, and discussions but lacked detailed titles, theoretical frameworks, and methodological rigor, especially in addressing bias and missing data, with mixed-method studies providing less detail on qualitative measures. The full risk of bias assessment for each study can be found in Supplementary Material E.

## Discussion

### Initiatives vary in objectives, format, and target population

Initiatives were predominately U.S. based, targeted towards underrepresented in medicine (URiM) and rural populations with the goals of increasing healthcare workforce diversity and rural healthcare workforce development. The overrepresentation of U.S.-based initiatives may stem from policy-driven efforts to increase healthcare workforce diversity [82], while the lack of European literature could be due to a focus on immediate workforce retention or due to the educational structure across most European nations, in which several systems consist of compulsory education followed by choice of educational tracks around age 15, usually between vocational and more academic paths [83]. This early career direction choice may result in a more integrated approach to career education being embedded earlier within school systems.

Concerning *objectives*, most initiatives focused on careers in nursing and medicine, reflecting a primary emphasis on these fundamental healthcare fields. However, many included specialized roles such as pharmacy, physical therapy and medical technologist positions. The diversity of career focus in these initiatives indicates an effort to broaden students' exposure to various healthcare pathways, yet the absence of certified nursing assistants (CNA) and healthcare aid professions is a notable gap, with only one publication specifically mentioning geriatric healthcare workers [71]. This is particularly relevant given increasing demand for long-term care workers and the ageing population requiring more comprehensive care [2]. The limited inclusion of these roles may reflect broader societal perceptions of these professions as less desirable careers, which may contribute to their underrepresentation in career promotion initiatives. Given the high turnover and workforce shortages in these professions [84], greater attention to their inclusion in early career initiatives may be warranted to challenge perceptions and ensure a well-rounded healthcare workforce.

Concerning the *format*, initiatives consistently highlighted the importance of hands-on activities and the inclusion of healthcare professionals in program delivery as suggested by various career theories and policy recommendations [15, 85]. Curricular design varied, ranging from disease-based approaches to medical scenario integration. Integrating medical simulations in the curriculum allows students to understand healthcare processes, from time of injury/symptom to diagnosis and treatment. They further allow students to recognize the professions that may be involved at each stage of care, while also educating students about specific diseases and injuries. Both approaches, along with body system designs and community health/health disparity initiatives, are valuable for their two-fold benefits. They increase awareness of direct patient care healthcare careers and enhance student health literacy, in terms of both procedural knowledge of healthcare systems and personal health. These benefits can lead to improved health outcomes and reduced unnecessary healthcare spending in the future.

As virtual and hybrid forms of delivery become more common, initiatives like *The Saturday Academy* [36] and *MAYO Clinic CARES* [74] suggest that digital and technology-enabled formats can achieve effectiveness comparable to in-person programs. The Saturday Academy is a 14-week weekly afterschool program designed to expose students to the field of dentistry, delivered fully virtually during the COVID-19 pandemic. The Mayo Clinic CARES program was also delivered during the COVID-19 pandemic in either hybrid or fully virtual formats, typically consisting of a 1-week enrichment camp followed by distance mentoring throughout the subsequent academic year. These approaches may expand reach to underserved populations by reducing geographic, logistical, and resource-related barriers; however, their implementation must account for persistent challenges related to the digital divide, including unequal access to reliable internet, devices, and digital literacy [86, 87]. In addition, successful adoption of virtual formats requires attention to infrastructure reliability, data privacy and security, availability of IT personnel, and ongoing troubleshooting and technical support. A gap identified through this review is the limited exploration of emerging technologies for direct patient care career promotion. Among these, virtual reality represents a promising example of a technology-enabled approach that could enhance access, particularly for rural and Indigenous students, by offering immersive career exploration experiences that may help mitigate the extent to which physical barriers and systemic disparities restrict access to career exploration opportunities commonly faced by these populations [88, 89]. Indigenous youth still face more barriers in terms of educational opportunities, due to reservation distance from urban centers and facilities, reduced networks and resources outside of reservations and higher safety and discrimination concerns [89]. In light of these barriers, it was questioned whether enrichment camp models requiring travel to urban centers are an appropriate method for indigenous populations [53]. Although virtual reality is well established in healthcare professional training [90–93], its application for secondary-level career exploration remains under-studied, as also highlighted by the Organisation for Economic Co-operation and Development (OECD) [94].

Another important finding from this review concerns the heterogeneity of *target populations*. Many initiatives focused on increasing healthcare workforce diversity, particularly for URiM groups like African American and Hispanic/Latinx students. However, some racial and ethnic minorities, such as Indigenous, Arabic, and Muslim students, were often overlooked, and LGBTQ+ identities were only considered in one initiative [43]. In order to create a truly inclusive healthcare workforce, capable of reducing health disparities and discrimination, it is essential to broaden the scope of these initiatives to include these often overlooked groups, while also ensuring programs address the historical and structural barriers that often limit these individuals from entering the healthcare workforce in the first place. Initiatives that were committed to diversity included targeted recruitment, culturally responsive curricula, and mentorship opportunities, but some lacked genuine commitment, appearing superficially aligned with diversity goals—potentially seeking funding without implementing substantial measures, such as failing to report URiM participant statistics in evaluations [45, 54–56] or omitting targeted recruitment for URiM populations [33, 38, 50, 74]. While these initiatives aim to address disparities through this targeted support and resource provision, it is important to remember that they operate within broader structural and systemic inequities, meaning their impact may be limited by factors beyond the scope of program design.

Furthermore, most initiatives were voluntary, requiring applications of self-selecting populations of students. A lack of school-mandated programs was observed, and is felt to be a missed opportunity, as such programs can clarify career aspirations and equalize opportunities for disadvantaged students [95]. While voluntary initiatives attract students already interested in direct patient care careers, mandatory initiatives can increase exposure to a wider range of students, fostering broader interest.

In contrast to what is suggested by career theories about beginning career exploration early [14, 15, 96], most initiatives targeted the 11th and 12th grades (ages 16–18). Super’s Developmental Self-Concept Theory suggests that direct patient care healthcare career initiatives should focus on early grades (7th to 9^th^, ages 12–15) to build knowledge and positive attitudes, laying the foundation for vocational self-concepts, which refers to an individual’s perception of their abilities, interests, and identity in relation to career choices, before students enter the exploration phase in high school. Initiatives targeting youth in the growth and early exploration stages should therefore be mandatory, ensuring all students develop vocational interests, while resources are allocated efficiently to maximize impact, particularly for students who may lack exposure to these careers. Considering the theoretical evidence, it may be too late for students in 11th and 12th grade to fully explore and develop their knowledge and interests in direct patient care careers. This aligns with the initiative’s evaluations results, where a large portion of reviewed studies [32, 41, 43, 46, 47, 51, 63, 65, 79] reported participants having pre-existing interest in direct patient care careers, suggesting that interest is formed earlier.

### Initiatives influence knowledge, attitudes, and career pathways

Overall, initiatives promoting direct patient care career pathways, regardless of objective or format, were successful in increasing knowledge, changing attitudes, and influencing career pathways. While programs were generally effective in maintaining or reinforcing student interest, they were not always successful in increasing it. Of all the assessed outcomes, interest most frequently saw non-significant changes post-program, with several publications attributing this to a possible ceiling effect where students already had high levels of interest prior to program participation [32, 35, 62].

Yet, interest, attitude, knowledge and career pathway outcomes are interrelated, which implies that direct patient care career interventions for secondary-level students often aim to address multiple facets of career exploration simultaneously. This interrelationship underscores the applicability of the *Adapted MOA Model* (Figure 1), which provides a useful framework for understanding how these variables interact and contribute to shaping career pathways. For example, the healthcare diversity summer enrichment camp for grades 10–12 provided a 5-day, campus-based voluntary program where students engaged with healthcare professionals and students across pharmacy, nursing, and dental medicine through lectures, simulations, laboratory activities, and campus tours, while also learning about admissions pathways, financial aid, and career opportunities. This program reported positive outcomes in interest, knowledge, and career pathway intentions [38].

The long-term impact of initiatives, such as choosing a healthcare career pathway or sustained interest in direct patient care careers, has remained to be comparatively under investigated. Several authors highlight that this gap is largely due to practical constraints, including limited funding [36, 69, 73], human and infrastructural resources [43, 53, 76], and the absence of mechanisms to track participants over time [33, 43, 44, 50, 69, 75]. Without these resources, long-term assessments are challenging, particularly for hard-to-reach populations targeted by many rural and URiM programs. To mitigate these challenges, establishing participant tracking mechanisms at the onset of programs is recommended, with surveys that allow for participant matching identified as a feasible method for follow-up [38, 64, 72, 73]. In settings with limited resources, highly resource-intensive evaluation methods are generally discouraged, highlighting the need for practical and sustainable tracking strategies. As a result, it remains unclear whether short-term outcomes, such as increased interest or improved attitudes, translate into actual entry into, or retention within, healthcare career pathways.

### The success of initiatives depends on contextual factors

The success of initiatives was influenced by policy landscapes, community involvement, funding, partnerships, and flexibility. Policy landscapes shaped funding availability and institutional support, often prompting local advocacy efforts and organizational backing to sustain initiatives. Engaging community stakeholders and demonstrating tangible, long-term benefits fostered sustained support, particularly in rural or medically underserved areas. While funding remains crucial, strategic planning and community involvement can enable initiatives to thrive even with limited resources [69, 76]. Beyond establishing partnerships, success requires a deep understanding of partner priorities, policies, and operational frameworks to align efforts effectively. Ultimately, cultivating strong, strategic partnerships and maintaining flexibility in initiative design and implementation are equally—if not more—critical than funding, ensuring initiatives can adapt to diverse needs and drive long-term impact.

However, the potential impact of increased early interest must be interpreted within broader contextual and system-level factors, including existing healthcare workforce shortages and downstream training capacity constraints, including limited professional school seats, clinical placement availability and levels of government and institutional support for expanding training capacity. While most included initiatives were delivered through universities, colleges, and/or in partnership with healthcare institutions, suggesting strong integration within existing training environments; this does not eliminate broader system-level capacity constraints. As a result, increased participation in such initiatives may still lead to increased competition for entry into professional programs. Such competition is not inherently negative and may support the selection of highly motivated candidates, provided workforce expansion occurs in parallel. These realities may also be usefully acknowledged within program design, for example, by preparing students for competitive selection processes and potential rejection as part of career development support.

The SEM [15] can guide direct patient care career initiative development by emphasizing the importance of considering these multiple contextual factors of influence. Effective initiatives should integrate microsystems such as peer groups, family, schools, and workplaces, while also considering exosystems like geographic location and socio-economic status to ensure accessibility. Additionally, broader macrosystem considerations, such as inclusive representation and retention strategies, should be incorporated to address issues like healthcare workforce shortages and to align with generational preferences, especially for digital natives like Generation Z and Alpha. Initiatives should incorporate technology and flexible learning environments to enhance engagement and appeal to the evolving preferences of future healthcare professionals.

### Towards an integrated model to guide initiatives promoting direct patient care healthcare career pathways

**FIGURE 4:**
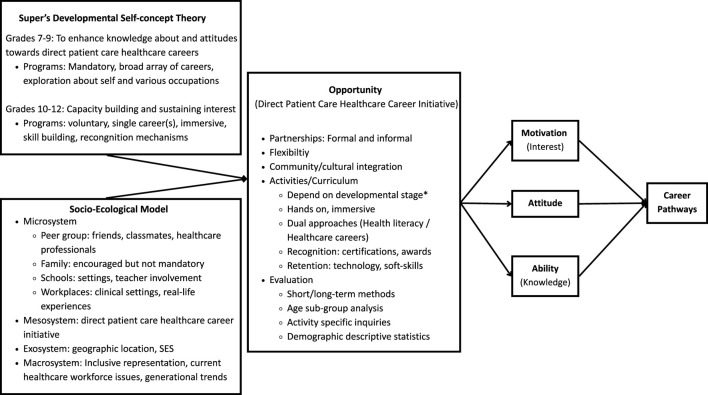
Integrated MOA-Informed Model (Lugano, Switzerland, 2024). Presents the Integrated Motivation–Opportunity–Ability informed model, which synthesizes the review findings by mapping key initiative characteristics identified in the literature onto each component of the career development process, illustrating how opportunity—shaped by developmental and socio-ecological considerations—relates to youth knowledge, interest, and attitudes and ultimately healthcare career pathways.

The *Integrated MOA-Informed Model* (Figure 4) suggests that incorporating the review’s findings—along with developmental-stage-appropriate activities and socio-ecological influences—into initiative design can enhance youth knowledge, interest, and attitudes toward direct patient care careers, and ultimately healthcare career pathways. Specifically, Super’s Developmental Self-Concept Theory [14] informs age-appropriate engagement strategies, and the SEM [15] highlights the impact of environmental influences (peers, schools, workplaces, and broader systems). Together, these elements create a structured approach recommended for designing future sustainable and impactful initiatives that influence healthcare career pathways.

### Limitations

The conclusions drawn from this review are hampered by some limitations that need to be acknowledged. The predominance of studies from North America limits the generalizability of the findings particularly regarding the educational systems of other countries and applicability to low-middle income countries. Differences in education systems and workforce policies may influence both the implementation and relevance of such initiatives across regions. Educational structures shape when and how career exposure occurs, and policy priorities influence which strategies are funded (e.g., long-term early recruitment strategies versus short-term workforce needs) and shape the goals and design of early career initiatives. As a result, variation in both policy contexts and educational structures across regions may limit the direct applicability of these initiatives in other settings.

There is also a lack of standardized outcome measurement and theory-based literature, along with limited subgroup analysis based on participant age or grade, restricting our understanding of how different age groups are impacted. Additionally, the review excluded non-English publications and was conducted by a single screener with a North American perspective. Future research should address these limitations by exploring primary school programs, age-specific impacts, long-term success, including retention within healthcare professions in addition to initial entry, and the integration of new technologies like virtual reality and social media. Investigating program influence on health literacy, would also be valuable, as would further exploring the relationship between program outcomes and career pathways, particularly by controlling for pre-existing interest and age factors.

## Conclusion

This systematic review examined the implementation and effectiveness of initiatives promoting direct patient care healthcare career pathways for secondary-level students by evaluating their impact on knowledge, interest, attitudes, and career pathways. The review included 50 publications and identified several key findings, such as the lack of non-North American representation, underrepresentation of essential healthcare roles, and underutilization of technology for broader outreach. While the available evidence suggests that such initiatives show promise, particularly when aligned with students’ developmental stages and socio-ecological contexts, the current evidence base is moderate in quality, geographically narrow, and heterogeneous in design. These limitations warrant a cautious interpretation of findings and restrict conclusions regarding long-term effectiveness and generalisability. The *Integrated MOA-Informed Model* is therefore proposed as a conceptual framework to guide the design and evaluation of future initiatives, rather than as a prescriptive or definitive model. Moving forward, there is a need for higher-quality and more geographically diverse studies, the development of standardized assessment tools, increasing mandatory program participation and greater attention to longitudinal outcomes. Leveraging digital and hybrid delivery formats may expand reach and inclusivity but must be implemented alongside rigorous evaluation strategies to strengthen the evidence base and inform sustainable, effective direct patient care career promotion initiatives.
