# Supplementary material for: Systematic review of initiatives promoting health career paths for secondary-level students
Source: Public Health Rev. 2026 Jul 2;47:1609646. doi: 10.3389/phrs.2026.1609646 (PMC13372724; doi:10.3389/phrs.2026.1609646)
Supplement: Supplementary file 1 [file Supplementaryfile1.docx]

**Manuscript prepared for Public Health Reviews**

**Systematic Review of Initiatives Promoting Health Career Paths for Secondary-level Students**

**Supplementary Materials**

**File A:** Database search string

Complete database search strategies used for the identification of relevant publications.

| Database | Search Query | Results (n) |
| --- | --- | --- |
| EBSCOhost (CINAHL, Communication & Mass Media Complete, Psychology and Behavioral Sciences Collection , APA PsycINFO | TI (High school* OR secondary school* OR middle school*) AND TI (intervention OR program* OR initiative OR education* OR promotion OR outreach OR curriculum) AND TI (Health* OR medical OR clinical OR nurs*) AND TI (career* OR job* OR profession* OR occupation*) | N = 48 |
| ProQuest (ERIC) | Title(High school* OR secondary school* OR middle school*) AND title(intervention OR program* OR initiative OR education* OR promotion OR outreach OR curriculum) AND title(Health* OR medical OR clinical OR nurs*) AND title(career* OR job* OR profession* OR occupation*)  Filters: Publication date from 2000 to 2023 | N = 11 |
| Web of Science | (High school* OR secondary school* OR middle school*) AND (intervention OR program* OR initiative OR education* OR promotion OR outreach OR curriculum) AND (Health* OR medical OR clinical OR nurs*) AND (career* OR job* OR profession* OR occupation*) | N = 40 |
| PubMed Advanced (PubMed, Medline) | ((((High school*[Title/Abstract] OR secondary school*[Title/Abstract] OR middle School*[Title/Abstract]) AND (career*[Title/Abstract] OR job*[Title/Abstract] OR profession*[Title/Abstract] OR occupation*[Title/Abstract])) AND (Health*[Title/Abstract] OR medical[Title/Abstract] OR clinical[Title/Abstract] OR nurs*[Title/Abstract])) AND (intervention[Title/Abstract] OR program*[Title/Abstract] OR initiative[Title/Abstract] OR education*[Title/Abstract] OR promotion[Title/Abstract] OR outreach[Title/Abstract] OR curriculum[Title/Abstract])) NOT (review*[Title/Abstract] OR meta-analysis[Title/Abstract]) AND (2000:2024[pdat]) | N = 2139 |

**File B:** Adapted Risk of Bias Checklist

Adapted quality assessment criteria used to evaluate included studies.

| MM Total | **44** |  |  |
| --- | --- | --- | --- |
| Quali Total | **34** |  |  |
| Quanti Total | **28** |  |  |
|  |  |  |  |
|  |  |  |  |
| **Topic** |  | **Checklist Item Description** | **Study** |
| **Title** | **1** | Includes study design and intervention type |  |
| **Abstract** | **2.1** | Informative and balanced summary of what was done and what was found (outcomes) |  |
|  | **2.2** | Program name with short description |  |
| **Introduction** | **3.1** | Explain the scientific background and rationale |  |
|  | **3.2** | State specific aims/objectives, research question(s) and any prespecified hypotheses |  |
| **Program Description** | **4.1** | Comprehensive description of program objectives, target population, duration/timeline, components and activities |  |
|  | **4.2** | Mentions specific HCCs |  |
|  | **4.2** | Theoretical framework guiding program design |  |
| **Methods** |  |  |  |
| Study design | **5.1** | Methodological orientation underpinning the study (grounded theory, ethnography, content analysis, discourse analysis, frequency analysis…) |  |
|  | **5.2** | Key elements of study design (pre/post, retrospective, pilot) |  |
| Setting | **5.3** | Describe the setting locations, and relevant dates (periods of recruitment, exposure, follow-up, and data collection) |  |
| Participants | **5.4** | Sampling methods/selection, program eligibility criteria, non-participation (school selection, community etc) |  |
|  | **5.5** | Method of approach (how were participants approached): face-to-face, phone, mail, email |  |
|  | **5.6** | Demographics (of program participants) (ages, grades, schools, locations) |  |
| Variables | **5.7** | Clearly define all outcomes and rationale for choice |  |
|  | **5.8** | Ethical review statement |  |
| Data collection & measurement | **5.9** | Details on data collection: Interview guides/prompts/questions provided? repeat interviews, audio/visual recording, field notes |  |
|  | **5.10** | Duration of FGs/interviews (NA for survey) |  |
|  | **5.11** | Was data saturation discussed? Were transcripts returned |  |
|  | **5.12** | Details of methods of measurement (type, validity, sample of survey items) |  |
| Bias | **5.13** | Describe any efforts to reduce sources of bias |  |
|  | **5.14** | Personal characteristics of interviewer/facilitator included (credentials, occupation, gender, experience/training) (NA for survey) |  |
|  | **5.15** | Relationship with participants (was a relationship established prior, what did participants know about the researcher (NA for survey) |  |
|  | **5.16** | What details of the interviewer/researcher were provided (biases, assumptions, reasons and interest in topic) |  |
| Data analysis | **5.17** | Describe statistical methods used |  |
|  | **5.18** | Explain how missing data was addressed |  |
|  | **5.19** | Number of coders |  |
|  | **5.20** | Derivation of themes (in advance or derived from data) |  |
|  | **5.21** | Software |  |
|  | **5.22** | Participant checking (did participants provide feedback on the findings) (NA for survey) |  |
| **Results** | **6.1** | Quotations presented to illustrate themes/findings |  |
|  | **6.2** | Consistency between the data presented and the findings |  |
|  | **6.3** | Clarity of major themes |  |
|  | **6.4** | Clarity of minor themes (description of diverse cases) |  |
| Descriptive data | **6.5** | Descriptive data of study participants provided (separated for cases/controls) |  |
|  | **6.6** | Tables Graphs |  |
| Outcome data | **6.7** | Data/numbers reported |  |
| Main results | **6.8** | Precision (confidence interval, level of significance) included |  |
| Other analysis | **6.9** | Report other analyses done (analyses of subgroups) |  |
| **Discussion** | **7.1** | Summarize key results with reference to study objectives |  |
|  | **7.2** | Discuss limitations - considering potential bias or imprecision & magnitude of potential bias (selection, self-reporting, confounding) |  |
|  | **7.3** | Cautious overall interpretation of results considering objectives, limitations, multiplicity of analyses, results from similar studies, and other relevant evidence (do they misrepresent their findings/outcomes?) |  |
|  | **7.4** | Discuss generalizability (external validity) |  |
| **Other** | **8.1** | Funding |  |

**File C: Summary of Individual Program Characteristics**

Detailed characteristics of included initiatives, including program structure, duration, setting, coordinating organizations, target populations, and key design features.

| Author, Year | Length | Sessions | Coordinated By | Setting | Attd. | Singular Career Focus | Body System Design | Community Health Education Embedded in Design | Virtual | Application | Fee | | Rec’t | | Parental Invol’t | | HCP Invol’t | | HCP Student Invol’t | |
| --- | --- | --- | --- | --- | --- | --- | --- | --- | --- | --- | --- | --- | --- | --- | --- | --- | --- | --- | --- | --- |
| Atance, 2018 | 1 week | - | U/C | Campus | Vol | X | X |  |  |  |  | X | |  | | X | | X | |  |
| Banuelos, 2016 | 2 weeks | - | U/C | Multi-site | Vol | X |  |  |  | X | X |  | |  | | X | |  | |  |
| Berk, 2014 | 13 weeks | weekly | U/C | School | Sch-mand |  |  |  |  |  |  |  | |  | | X | |  | |  |
| Briskey, 2017 | 3 weeks | - | U/C | Campus | Vol | X |  |  |  | X |  |  | |  | | X | |  | |  |
| Burns, 2019 | 14 weeks | biweekly - 3 hours | U/C | Campus | Vol | X |  |  |  | X |  | X | | X | | X | | X | |  |
| Burns, 2021 | 14 weeks | biweekly - 3 hours | U/C | Online | Vol | X |  |  | X | X |  |  | | X | | X | |  | |  |
| Butler, 2022 | 5 days | daily - 7 hours | U/C | Campus | Vol |  |  |  |  | X |  |  | |  | | X | | X | |  |
| Coffin, 2022 | 1 day | - | - | Campus | Vol | X |  |  |  |  |  | X | |  | | X | |  | |  |
| Crawford, 2019 | - | 3 sessions / year | Gov | School | Vol |  |  | X |  |  |  |  | |  | | X | |  | |  |
| Crump, 2014 | 3 weeks | - | U/C | Multi-site | Vol |  |  |  | X |  |  | X | |  | | X | |  | |  |
| D'anna, 2019 | 1 semester - 1 academic year | - | U/C | School | Vol |  |  |  |  |  | X |  | |  | |  | |  | |  |
| Das, 2023 | 1/2 day | - | U/C | School | Sch-mand | X |  | X |  |  |  |  | |  | | X | | X | |  |
| Dicosmo, 2021 | 1 day | 4 hours | U/C | Campus | Vol | X |  |  |  | X |  | X | | X | | X | | X | |  |
| Fernandez-Repolle, 2018 | 1 week | Monthly mentorship sessions X9 months | U/C | Multi-site | Vol |  |  |  | X |  |  | X | |  | | X | | X | |  |
| Freischlag, 2019 | 1 semester | 10 sessions X 90 minutes | - | School | Vol | X |  |  |  |  |  |  | |  | | X | | X | |  |
| Frey, 2021 | 1 day | - | U/C | Campus | Vol | X |  |  |  |  |  | X | |  | | X | | X | |  |
| Gefter, 2018 | 3 years | 12-15 sessions / year | - | School | Vol |  |  | X |  |  |  |  | |  | | X | | X | |  |
| Ghazali, 2020 | 1 day | 80 minutes |  | School | Vol | X |  |  |  |  |  |  | |  | | X | |  | |  |
| Goldsmith, 2014 | 1/2 day | - | U/C | Campus | Vol | X |  |  |  | X |  | X | |  | | X | | X | |  |
| Gómez, 2018 | 6 weeks | - | Hospital | Hospital | Vol | X |  |  |  | X |  | X | |  | | X | |  | |  |
| Hamrick, 2019 | 1 week | daily - 8 hours | U/C | Campus | Vol | X | X |  |  | X |  | X | |  | |  | | X | |  |
| Henderson, 2015 | 1/2 day | 3 - 4 sessions / year | U/C | Campus |  | X |  |  |  |  |  |  | |  | | X | | X | |  |
| Herek, 2019 | 1 day | - | U/C | Comm | Sch-mand |  |  | X |  |  |  |  | |  | |  | | X | |  |
| Holden, 2015 | 10 weeks | weekly | - | School | Vol |  | X | X |  |  |  |  | | X | | X | | X | |  |
| Holden, 2013 | 2 weeks | 10 sessions, 3 hours | - | Online | Vol |  | X | X | X |  |  | X | |  | | X | |  | |  |
| Inglehart, 2014 | 15 weeks | weekly | U/C | Multi-site | Vol | X |  | X |  |  |  | X | | X | |  | | X | |  |
| Kadavakollu, 2020 | 5 weeks | weekdays, 6 hours/day | U/C | Campus | Vol | X |  |  |  | X |  | X | |  | | X | | X | |  |
| Karpa, 2015 | 13 weeks | weekly, 2 hours | U/C | - | Vol |  | X |  |  | X |  | X | |  | | X | | X | |  |
| Kaye, 2014 | 1 day | 5.5 hours | U/C | Campus | Vol | X | X |  |  | X |  |  | |  | |  | | X | |  |
| Kendrick, 2020 | 5 weeks | 14 sessions; 2 -3 sessions / week |  | Hospital | Vol |  |  |  |  | X |  |  | |  | | X | |  | |  |
| Keselman, 2015 | - | - | School | School |  |  |  | X |  |  |  |  | |  | |  | |  | |  |
| Keselman, 2019 | **-** | 19 lessons | - | School |  |  |  | X |  |  |  |  | |  | |  | |  | |  |
| Kohut, 2023 | 6 years | Academic year -weekly July - weekdays | Multiple | Multi-site | Vol |  | X | X |  | X |  |  | |  | | X | | X | |  |
| Kumar, 2015 | 1 day | - | U/C | - | Vol |  |  | X |  |  |  |  | |  | | X | | X | |  |
| Labadie, 2017 | 2 weeks | - | U/C | Hospital | Vol | X |  |  |  | X | X |  | |  | |  | | X | |  |
| Macaskill, 2023 | 1 day | 6 hours | Gov | Hospital | Vol |  |  |  |  |  |  |  | |  | | X | | X | |  |
| Maurice, 2019 | 1 day | - | U/C | School | Sch-mand |  |  |  |  |  |  |  | |  | |  | | X | |  |
| Mayberry, 2018 | 2 years | 4 sessions | U/C | Multi-site | Vol | X |  | X |  |  |  |  | |  | | X | | X | |  |
| Oshiro, 2023 | variable | variable | U/C | Online | Sch-mand |  |  |  | X |  |  |  | |  | |  | | X | |  |
| Patel, 2017 | - | 8 sessions. 45-50 minutes | - | School | Vol |  |  | X |  |  |  |  | |  | |  | |  | |  |
| Patel, 2015 | variable | ~2.5 hours/week | U/C | Campus | Vol | X |  |  |  | X |  | X | |  | |  | | X | |  |
| Pezzullo, 2023 | 14 weeks | biweekly - 3 hours | U/C | Multi-site | Vol | X |  |  |  | X |  |  | |  | |  | | X | |  |
| Pruszynski, 2022 | In-person: 5 weeks Virtual: 1 week | 3 days/week | U/C | Campus | Vol |  |  |  | X | X |  | X | | X | | X | |  | |  |
| Robinson, 2017 | 1 day | - | U/C | School | Sch-mand | X |  |  |  |  |  | X | |  | |  | | X | |  |
| Rocha, 2022 | 3 years | 4 weeks/annually | Multiple | Multi-site | Vol |  | X |  |  | X |  |  | |  | | X | |  | |  |
| Rodriguez, 2022 | 1 year | 5 sessions | - | - | Vol | X |  | X |  |  |  | X | |  | |  | | X | |  |
| Rogers, 2021 | 1 day | - | - | - | Vol | X |  |  |  | X |  | X | |  | | X | | X | |  |
| Shaikh, 2013 | 4 days | - | Gov | Hospital | Vol | X |  |  |  |  |  |  | |  | |  | |  | |  |
| Tawash, 2018 | - | - | U/C | School |  | X |  |  |  |  |  |  | |  | |  | | X | |  |
| Zhang, 2016 | 1 day | 6 hours | U/C | Campus | Sch-mand |  | X |  |  |  |  |  | |  | |  | | X | |  |

* ‘-’ indicates no information was provided

*Invol’t = involvement, rec’t = recruitment

*U/C = university or college, GOV = government organization, Comm = community, Vol = voluntary, Sch-mand = school mandatory

**File D: Summary of Individual Program Activities**

Detailed descriptions of activities and learning experiences incorporated within each included initiative.

| Author, Year | Mentoring | Tours / Field Trip | Hands-On Work Shops/ Labs | Simulation / Mock Procedures | Medical CBL | Lectures / Didactics | Internship / Shadowing | Credits/ Cert | Final Project / Exam | Rsch | Final Ceremony | Profess Skills | Pathway Info Session | Prep | Career Panels / Counseling |
| --- | --- | --- | --- | --- | --- | --- | --- | --- | --- | --- | --- | --- | --- | --- | --- |
| Atance, 2018 |  | X | X | X |  | X |  |  |  | X |  |  |  |  | X |
| Banuelos, 2016 | X |  | X | X |  | X |  |  |  |  | X |  |  |  |  |
| Berk, 2014 |  |  | X | X | X | X |  | CREDIT |  |  |  |  |  |  |  |
| Briskey, 2017 |  |  | X | X | X | X | X | CPR |  |  |  |  |  |  |  |
| Burns, 2019 |  |  | X |  |  |  |  |  |  |  | X | X | X | X |  |
| Burns, 2021 |  |  | X |  |  |  |  |  |  |  | X | X | X | X |  |
| Butler, 2022 |  | X | X |  |  |  |  |  |  |  |  |  | X |  |  |
| Coffin, 2022 |  |  | X | X | X |  |  |  |  |  |  |  |  |  |  |
| Crawford, 2019 |  | X |  |  |  | X |  |  |  |  |  |  |  |  |  |
| Crump, 2014 |  | X | X |  |  | X | X | STIPEND CERT |  |  |  |  |  | X |  |
| D'anna, 2019 |  |  |  |  |  | X | X | CREDIT |  |  |  |  |  |  |  |
| Das, 2023 |  |  | X |  |  | X |  |  |  |  |  |  |  |  | X |
| Dicosmo, 2021 |  |  | X | X |  | X |  |  |  |  |  |  | X |  |  |
| Fernandez-Repolle, 2018 | X |  | X |  |  |  |  |  |  |  |  |  |  |  |  |
| Freischlag, 2019 |  | X | X |  | X | X |  | CERT | X |  |  |  |  |  |  |
| Frey, 2021 |  | X | X |  |  | X |  |  | X |  | X |  |  |  | X |
| Gefter, 2018 | X | X | X |  | X |  |  |  |  |  |  |  |  |  | X |
| Ghazali, 2020 |  |  |  |  |  | X |  |  |  |  |  |  | X |  |  |
| Goldsmith, 2014 |  |  | X |  |  |  |  |  |  |  |  |  |  |  | X |
| Gómez, 2018 |  |  | X |  |  |  | X | STIPEND | X |  |  |  |  |  |  |
| Hamrick, 2019 |  | X | X |  |  | X |  |  |  |  |  |  |  |  |  |
| Henderson, 2015 | x |  | X | X |  |  |  |  |  |  |  |  |  |  |  |
| Herek, 2019 |  |  | X |  | X |  |  |  |  |  |  |  |  |  |  |
| Holden, 2015 |  | X | X | X |  | X |  | CPR | X |  |  |  |  | X |  |
| Holden, 2013 |  |  |  |  |  | X |  | CERT | X |  |  | X |  |  |  |
| Inglehart, 2014 | X | X | X | X |  | X |  | CREDIT HIPAA | X |  | X |  |  |  |  |
| Kadavakollu, 2020 |  | X | X | X |  |  |  | CPR FA |  |  |  |  | X | X | X |
| Karpa, 2015 | X |  | X |  | X | X | X | CREDIT | X |  |  |  |  |  | X |
| Kaye, 2014 |  |  | X | X | X |  |  |  |  |  |  |  |  |  |  |
| Kendrick, 2020 | X |  | X |  |  |  | X |  |  |  |  |  |  |  |  |
| Keselman, 2015 | X |  |  | X |  | X |  |  | X |  |  | X |  |  |  |
| Keselman, 2019 |  |  |  |  |  | X |  |  |  |  |  |  |  |  |  |
| Kohut, 2023 | X | X | X |  | X | X | X | STIPEND |  |  |  | X |  | X | X |
| Kumar, 2015 |  | X |  |  |  |  |  |  |  |  |  |  | X |  |  |
| Labadie, 2017 | X |  | X |  | X | X | X | BLS CREDIT | X |  | X | X | X |  |  |
| Macaskill, 2023 |  | X | X |  | X |  |  | CPR |  |  |  |  |  |  |  |
| Maurice, 2019 |  |  | X |  |  |  |  |  |  |  |  |  |  |  |  |
| Mayberry, 2018 |  | X | X |  |  |  |  |  |  |  |  |  | X |  | X |
| Oshiro, 2023 | X |  |  |  |  |  |  |  |  |  |  |  |  |  |  |
| Patel, 2017 |  |  | X |  |  | X |  |  |  |  |  |  |  |  |  |
| Patel, 2015 | X |  |  |  | X |  | X | CREDIT |  |  |  |  | X |  |  |
| Pezzullo, 2023 |  |  | X |  |  |  |  |  |  |  | X | X | X | X |  |
| Pruszynski, 2022 | X (LT) | X | X |  |  |  |  |  |  |  | X | X |  | X |  |
| Robinson, 2017 | X (LT) |  | X |  |  | X |  |  |  |  |  |  | X |  |  |
| Rocha, 2022 | X | X | X | X |  | X |  |  |  |  |  |  |  | X |  |
| Rodriguez, 2022 | X |  | X |  |  |  |  | CERT | X |  |  |  | X | X |  |
| Rogers, 2021 |  |  | X |  | X |  |  |  |  |  |  |  | X |  |  |
| Shaikh, 2013 |  |  | X | X | X | X |  |  |  |  |  |  |  |  |  |
| Tawash, 2018 |  |  | X |  |  |  |  |  |  |  |  |  |  |  | X |
| Zhang, 2016 |  |  | X |  | X | X |  |  |  |  |  |  |  |  |  |

* CBL = Case based learning, RSCH = research, Prep = preparatory

*LT = long-term, CERT = certification, BLS = basic life support, FA = first aid, CPR = cardiopulmonary resuscitation

**File E: Quality Assessment for Each Individual Study**

Results of the quality assessment conducted for each included study using the adapted risk of bias checklist.

**
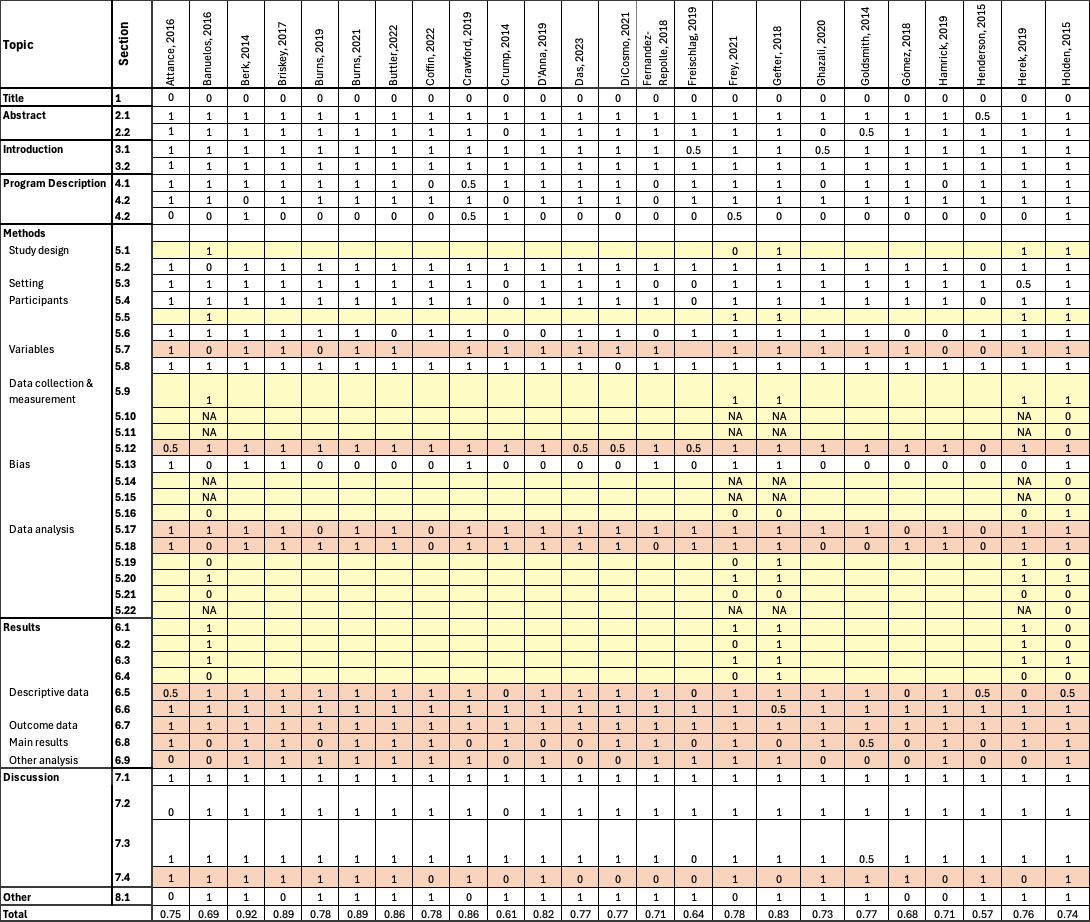
**

**
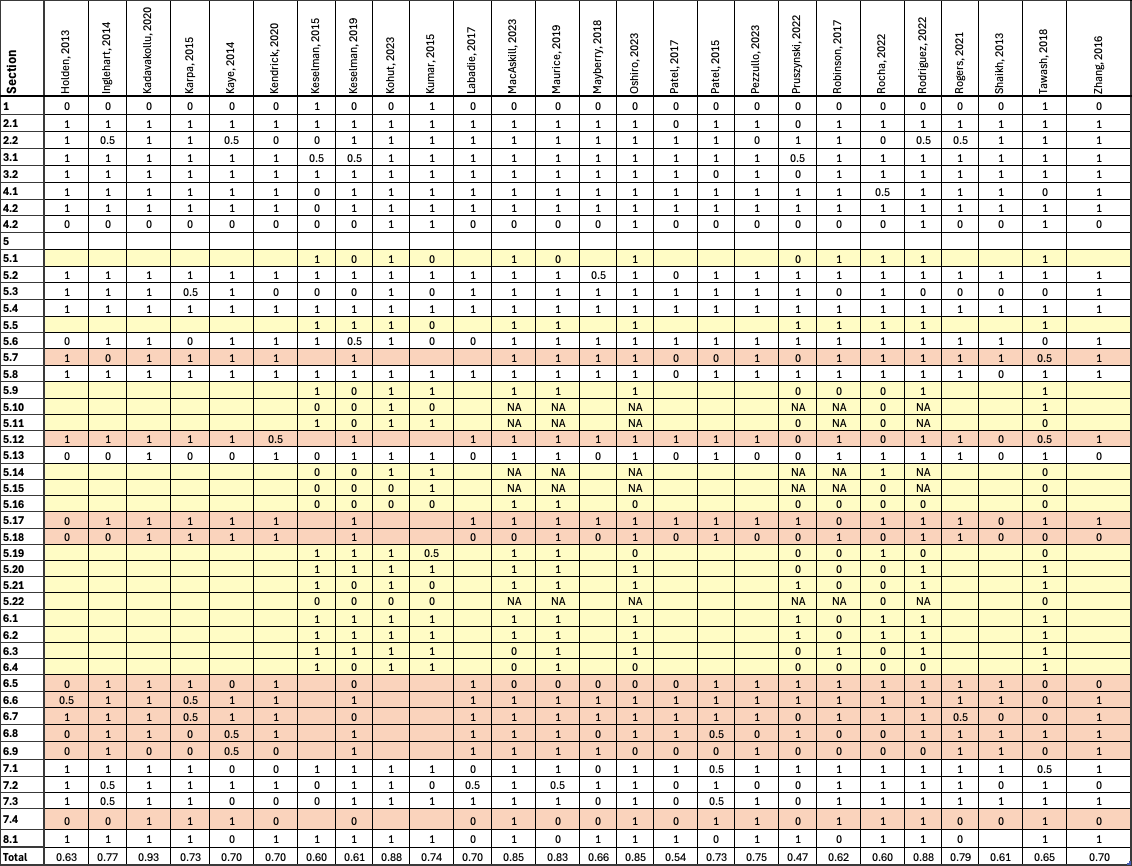
**
